# Recognition of 27-Class Protein Folds by Adding the Interaction of Segments and Motif Information

**DOI:** 10.1155/2014/262850

**Published:** 2014-07-21

**Authors:** Zhenxing Feng, Xiuzhen Hu

**Affiliations:** Department of Sciences, Inner Mongolia University of Technology, Hohhot, China

## Abstract

The recognition of protein folds is an important step for the prediction of protein structure and function. After the recognition of 27-class protein folds in 2001 by Ding and Dubchak, prediction algorithms, prediction parameters, and new datasets for the prediction of protein folds have been improved. However, the influences of interactions from predicted secondary structure segments and motif information on protein folding have not been considered. Therefore, the recognition of 27-class protein folds with the interaction of segments and motif information is very important. Based on the 27-class folds dataset built by Liu et al., amino acid composition, the interactions of secondary structure segments, motif frequency, and predicted secondary structure information were extracted. Using the Random Forest algorithm and the ensemble classification strategy, 27-class protein folds and corresponding structural classification were identified by independent test. The overall accuracy of the testing set and structural classification measured up to 78.38% and 92.55%, respectively. When the training set and testing set were combined, the overall accuracy by 5-fold cross validation was 81.16%. In order to compare with the results of previous researchers, the method above was tested on Ding and Dubchak's dataset which has been widely used by many previous researchers, and an improved overall accuracy 70.24% was obtained.

## 1. Introduction

With the accomplishment of the Human Genome Project, the “post genome era” has presented a large number of protein sequences that have challenged to develop a high-throughput computational method to structurally annotate the sequences coming from genomic data. One of these critical structures, the protein fold, reflects a key topological structure in proteins as it contains three major aspects of protein structure: the units of secondary structure, the relative arrangement of structures, and the overall relationship of protein peptide chains [1].

A protein can only perform its physiological functions if it folds into its proper structure. Abnormal protein folding causes molecular aggregation, precipitation, or abnormal transport, resulting in different diseases. For example, the prion protein (PRNP) accumulates in the brain and causes neurodegenerative diseases, such as scrapie, Creutzfeldt-Jakob disease, Parkinson's disease, Huntington's disease, and mad cow disease. And the PRNP is a pathogenic protein caused by the abnormal folding of proteins. Thus, the correct identification of protein folds can be valuable for the studies on pathogenic mechanisms and drug design [2–8]. Thus, the identification of folding types is a highly important research project in bioinformatics.

Proteins with similar sequences tend to fold into similar spatial structures. When proteins have close evolutionary relationships, the similarity-based methods can achieve very reliable predicted results [9, 10]. However, they are unreliable for the identification of proteins with far evolutionary relationships. Fortunately, approaches that extract relevant features from protein sequences to identify protein folds have made significant advance in recent years. In 2001, Ding and Dubchak [11] built the 27-class folds dataset and used multiple feature groups as parameters, including amino acid composition, predicted secondary structure, hydrophobicity, Van der Waals volume, polarity, and polarizability; then they proposed support vector machines and neural networks methods for the identification of 27-class protein folds. The best overall accuracy achieved was 56%.

Afterwards, based on the dataset built by Ding and Dubchak and the feature parameters, some researchers suggested algorithm improvements for the identification of folds. For example, Chinnasamy et al. [12] introduced the phylogenetic tree and Bayes classifier for the identification of protein folds and structural classifications and achieved an overall accuracy of 58.18% and 80.52%, respectively. Nanni [13] proposed a new ensemble of K-local hyperplane based on random subspace and feature selection and achieved an overall accuracy of 61.1%. Guo and Gao [14] presented a novel hierarchical ensemble classifier named GAOEC (Genetic-Algorithm Optimized Ensemble Classifier) and achieved an overall accuracy of 64.7%. Damoulas and Girolami [15] offered the kernel combination methodology for the prediction of protein folds and the best accuracy was 70%. Lin et al. [16] exploited novel techniques to impressively increase the accuracy of protein fold classification.

Based on the dataset built by Ding and Dubchak, other previous researchers suggested the selection of feature parameters for the identification of protein folds. For example, Shamim et al. [17] used the feature parameters of structural properties of amino acid residues and amino acid residue pairs and achieved an overall accuracy of 65.2%. Dong et al. [18] proposed a method called ACCFold and achieved an overall accuracy of 70.1%. Li et al. [19] proposed a method called PFP-RFSM and got improved results for the identification of protein folds.

Based on the dataset built by Ding and Dubchak [11], many pioneering researchers have not only focused on the selection of feature parameters but also on the improvement of algorithms to identify protein folds. For example, Zhang et al. [20] in our group proposed an approach of increment of diversity by selecting the pseudo amino acid composition, position weight matrix score, predicted secondary structure, and the second neighbor dipeptide composition; by using the characteristic parameters to predict 27-class protein folds and structural classifications, the overall accuracies measured up to 61.10% and 79.11%, respectively. Shen and Chou [21] applied the OET-KNN ensemble classifier to identify folding types by introducing pseudo amino acid with sequential order information as a feature parameter and achieved an overall accuracy of 62.1%. Chen and Kurgan [22] proposed the PFRES method using evolutionary information and predicted secondary structure, obtaining an accuracy of 68.4%. Ghanty and Pal [23] proposed the Fusion of Heterogeneous Classifiers approach, with features including the selected trio AACs and trio potential, and the overall recognition accuracy reached 68.6%. Shen and Chou [24] applied a method to identify protein folds by using functional domain and sequential evolution information and achieved an overall accuracy of 70.5%. Yang et al. [25] proposed a novel ensemble classifier called MarFold, which combines three margin-based classifiers for protein folds recognition, and the overall prediction accuracy was 71.7%.

Other previous researchers have constructed new 27-class fold datasets and performed corresponding researches. For example, based on the Astral SCOP 1.71, with sequence identity below 40%, Shamim et al. [17] constructed a dataset including 2554 proteins belonging to 27-class folds, proposed structural properties of amino acid residues and amino acid residue pairs as parameters, and achieved an overall accuracy of 70.5% by 5-fold cross validation. Based on the Astral SCOP 1.73 with sequence identity below 40%, Dong et al. [18] constructed the 27-class folds dataset which contains 3202 sequences, proposed an ACCFold method, and obtained an overall accuracy of 87.6% by 5-fold cross validation. According to Ding and Dubchak's [11] description on the construction of protein folds dataset in literature, based on the Astral SCOP 1.75, Liu and Hu [26] in our group constructed an expanded 27-class folds dataset. The dataset with a sequence identity below 35% contains 1895 sequences. Motif frequency, low-frequency power spectral density, amino acid composition, predicted secondary structure, and autocorrelation function values were combined as feature parameters to identify the 27-class protein folds and structural classifications, and the overall accuracy by independent test was 66.67% and 89.24%, respectively. At the same time, researches on 76-class folds, 86-class folds, and 199-class folds have also made some progress [18, 27].

In this paper, based on the dataset built by Liu et al. [27] in our group, amino acid composition, motif frequency, predicted secondary structure information, and the interaction of predicted secondary structure segments were applied for the recognition of protein folds. These features reflect the sequence information, structural information, and functional information. Based on the ensemble classification strategy with the Random Forest algorithm, improved identification results of 27-class protein folds and structural classifications were achieved.

## 2. Materials and Methods

### 2.1. Protein Folds Dataset

The dataset used in this paper was built by Liu et al. [27] in our group. The sequence identity of the dataset was below 35%. The number of sequences in each fold was not less than 10. The training set and testing set contained 956 protein chains and 939 protein chains, respectively. The distribution of corresponding folds name, number of sequences, and structural class is shown in Table 1. The other dataset used in this paper was built by Ding and Dubchak and reorganized by Shen and Chou [21]. The dataset with sequence identity below 35% used by many researchers contained a training set including 311 protein chains and a testing set including 383 protein chains. The distribution information of corresponding protein folds is also shown in Table 1. The dataset is available at the following website: http://202.207.29.245:8080/Ha/HomePage/fzxHomePage.jsp.

The dataset built in our group was according to Ding and Dubchak's description about the construction of protein folds dataset in literature [11]. The number of sequences in the dataset was three times greater than Ding and Dubchak's dataset [11].

### 2.2. The Selection of Feature Parameters

#### 2.2.1. Amino Acid Composition (A)

The distributions of the 20 amino acid residues in protein sequences for different protein folds are obviously different, and previous researches have shown that amino acid composition is associated with protein folding information [11, 15, 17, 22]. In this paper, we extracted the occurrence frequencies of 20 amino acid residues in protein sequences; thus we got a 20-dimensional vector. Thus amino acid composition was proposed as a feature parameter (A).

#### 2.2.2. Motif Information (M)

A motif is the conserved local region in a protein during evolution [28], which often has a relationship with biological functions. For example, some motifs are related to DNA-binding sites and enzyme catalytic sites [29]. As feature parameters, motif information has been successfully applied for the prediction of super family, protein folds, and so forth [27, 28, 30]. Two kinds of motifs were used in this paper: one with biological functions obtained by searching the existing functional motif dataset PROSITE [31] and the other with statistically significant motifs searched by MEME (http://meme.nbcr.net/meme/cgi-bin/meme.cgi). Motif information (M) includes functional motifs and statistical motif.

(*1) Functional Motif*. The PROSITE dataset was used to gather protein sequence patterns with notable biological functions. PS_SCAN packets provided by the PROSITE dataset and compiled by a Perl program were used as a motif-scan tool to search the sequences of 27-class folds training set; 45 functional motifs were obtained and selected. For an arbitrary sequence in dataset, the frequencies of different motifs in the sequence were recorded. If a motif occurred once, the corresponding frequency value was recorded as “1”; if the motif occurred twice, the value would be 2, and so on; otherwise if the motif did not occur, the corresponding frequency value was recorded as “0.” Thus, the frequencies of different functional motifs in a protein sequence were converted into a 45-dimensional vector.

(*2) Statistical Motif*. For statistical motifs, MEME was applied as the motif-scan tool. MEME has been widely used to search protein motifs and DNA sequences [32]. In this paper, the motifs with the three highest frequencies in each kind of folds were selected. Each motif contains 6–10 amino acid residues; thus 81 motifs were obtained from the 27-class folds training set. For an arbitrary sequence in dataset, when a motif occurred once, the frequency value was recorded as “1”; if the motif occurred twice, the value would be 2, and so on; otherwise if the motif did not occur, the corresponding frequency value was recorded as “0.” Thus, frequencies of different statistical motifs in a protein sequence were converted into an 81-dimensional vector.

#### 2.2.3. The Interaction of Segments (ACC)

A previous study showed that predicted secondary structure information is a main feature parameter for the identification of multiclass protein folds [11, 17, 21, 22]. Here, the online web server PSIPRED (http://bioinf.cs.ucl.ac.uk/psipred/) was used as the tool to obtain the predicted secondary structure of each protein sequence in the 27-class protein fold dataset. As a protein fold is a description based on the secondary structure, the interaction between secondary structures plays an important part on the folding of protein. In this paper, we extracted the average interactions between predicted secondary structure segments as a feature parameter for the recognition of protein folds.

(*1) The Calculation of Autocross Covariance (ACC)*. The autocross covariance (ACC) [33] has been successfully adopted by many researchers for the prediction of protein folds [18], G-proteins [34, 35], protein interaction predictions [36], and *β*-hairpins [37]. However, ACC has mainly been used on the research between residues or bases. In this paper, the ACC was used at the level of predicted secondary structure segments (helix, strand, or coil) for the first time to predict protein folds. ACC contains two kinds of variables: the AC variable measures the correlation of the same property (the same property means that two secondary structure segments are the same type) and the CC variable measures the correlation of the different properties. In this paper, given the corresponding predicted secondary structure segments (helix, strand, or coil) in one sequence, AC variables describe the average interactions in the same type of predicted secondary structure segments, and the separation distance between two predicted secondary structure segmentsis lg segments. For example, if two segments are neighboring, then lg = 1; if the two segments are next-to-neighboring, then lg = 2, and so on. The AC variables are calculated according to the following equation:

(1) $$\begin{array}{c} \mathrm{AC}\left(i,\text{lg}\right)=\overset{L-\text{lg}}{\underset{j=1}{\sum }}\frac{\left(S_{i,j}-{\overset{-}{S}}_{i}\right)\left(S_{i,j+\text{lg}}-{\overset{-}{S}}_{i}\right)}{\left(L-\text{lg}\right)}\;\left(i=1,2,3\right), \end{array}$$

where ${\overset{-}{S}}_{i}=\sum_{j=1}^{L}s_{i,j}/L$, *i* is one kind of secondary structure segment (helix, strand, or coil), *L* is the number of the secondary structure segments in a protein sequence, and *S*_*i*,*j*_ is a property value of secondary structure segment *i* at position *j*. ${\overset{-}{S}}_{i}$ is the average property value for segment *i* along the whole sequence.

For example, given the hydrophobicity values (Table 2) for 20 amino acid residues, the secondary structure segment *i* contains *m* residues, and *S*_*i*,*j*_ represents the summation of hydrophobic values for the *m* residues. The dimension of AC⁡ variables is 3 ∗max (lg).

**Figure 1:**
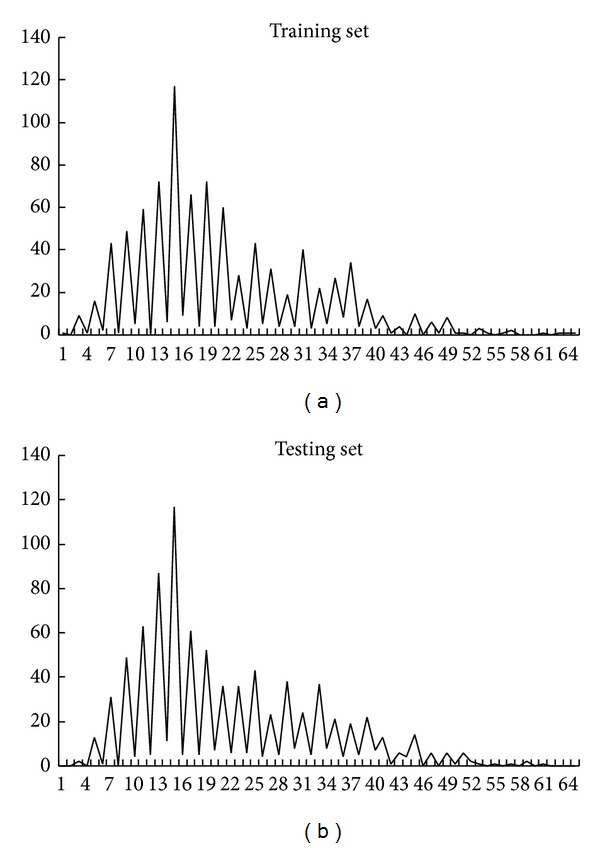
The numbers of sequences containing secondary structure segments. (a) and (b) are for training set and testing set, respectively.

CC variables describe the average interactions between different types of secondary structure segments, which can be calculated according to the following equation:

(2) $$\begin{array}{c} \text{CC}\left(i1,i2,\text{lg}\right)=\overset{L-\text{lg}}{\underset{j=1}{\sum }}\frac{\left(S_{i1,j}-{\overset{-}{S}}_{i1}\right)\left(S_{i2,j+\text{lg}}-{\overset{-}{S}}_{i2}\right)}{\left(L-\text{lg}\right)}, \end{array}$$

where *i*1 and *i*2 are two different types of secondary structure segments (helix, strand, or coil) and *S*_*i*1,*j*_ is a property value of secondary structure segment *i*1 at position *j*. ${\bar{S}}_{i1}({\bar{S}}_{i2})$ is the average property value for secondary structure segment *i*1(*i*2) along the whole sequence. The dimension of CC variables is 3∗2∗lg.

(*2) The Selection of the Maximum Value of lg*. The statistical analysis of the number of the secondary structure segments in the 27-class folds dataset is shown in Figure 1. The abscissa represents the number of secondary structure segments. The ordinate represents the number of sequences. The percentage of sequences that contained less than five secondary structure segments was below 0.5%. The maximum value of lg was selected as 4 (max (lg) = 4).

(*3) The Values of Physicochemical Properties*. In this paper, four physicochemical properties used by many researchers were selected as ACC feature values to calculate the interactions of segments: hydrophobicity (H1), hydrophilicity (H2), polarity (PL), and solvent accessible surface area (SASA). The values of physicochemical properties for the 20 amino acid residues were taken from the literature of Guo et al. [36] (Table 2). ACC can reflect the segments-order and the long-range correlation information of the sequence, which has a major influence on protein folding.

#### 2.2.4. Predicted Secondary Structure Information (P)

As the protein fold is a description based on the secondary structure, the formation of secondary structure in sequence influences the folding of protein. From the researches of published literatures [17, 21, 22, 25], in this paper, the occurrence frequencies of three kinds of predicted secondary structure segments motifs were extracted as a feature parameter; thus we can get a 3-dimensional vector. Then the occurrence frequencies of three-state of amino acid residues (i.e., helix, strand, and coil) were extracted as a feature parameter, and then we got another 3-dimensional vector. Therefore, the frequencies of secondary structure segments and three-state of amino acid residues above were converted into a 6-dimensional vector, which were represented by P. Here, the online web server PSIPRED (http://bioinf.cs.ucl.ac.uk/psipred/) was used to get the predicted secondary structure of each protein sequence.

### 2.3. Random Forest

Random Forest is an algorithm for classification developed by Breiman [38]. The basic idea of the algorithm is that multiple weak classifiers compose a strong individual classifier. Random Forest uses a collection of multiple decision trees, where each decision tree is a classifier, every split of the decision tree is a classifier, and the final predictions are made by the majority vote of trees. Random Forest has the following advantages: (1) few parameters to adjust and (2) data that does not require preprocessing. And Random Forest has two important parameters: (1) the number of feature parameters selected by each node of a single decision tree at each split, the number being represented by *m* ($m=\sqrt{M}$, where *M* is the total number of features which were selected initially), and (2) the number of decision trees represented by *k* (in this paper, *k* = 1000). The Random Forest algorithm has been successfully used for the prediction of antifreeze proteins [39], DNA-binding residues [40], the metabolic syndrome status [41], *β*-hairpins [42], and so forth. Here, R-2.15.1 software (http://www.r-project.org/) was used to perform the Random Forest algorithm by calling the randomForest program package.

## 3. Results and Discussion

### 3.1. The Comparison Results with Different Parameters

For the 27-class fold dataset, amino acid composition, motif frequency, predicted secondary structure information, and the interaction of secondary structure segments were extracted as feature parameters, with the combined feature vector as input parameters for the Random Forest algorithm. The overall accuracy of the testing set in the dataset measured up to 78.38% by independent test.

**Figure 2:**
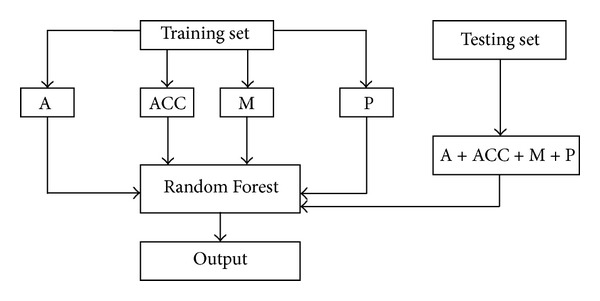
The architecture of the protein folds identification system.

For further comparison, identification results from the gradual addition of relevant feature parameters are listed (Table 3). Furthermore, the architecture of the protein folds identification system is shown (Figure 2).

When only one feature parameter, amino acid composition, was used, the overall accuracy was 43.66% (Table 3). After adding the feature parameter based on the interaction of segments, the overall accuracy increased to 68.80% (a 25% higher overall accuracy). The accuracies for folds 1, 2, 4, 6, 19, and 20 increased more than 50%, and the accuracies of folds 3, 11, 12, 13, 25, and 26 increased approximately 30% except folds 15 and 27, and the accuracies of the remaining folds also improved in different degrees. The feature parameter based on the interaction of segments also had a great effect on the identification of protein folds. When adding the feature parameter of motif frequency to amino acid composition and interaction of segments, the overall accuracy increased to 76.25%, an 8% higher overall accuracy. During this process, the accuracies of folds 12, 13, 19, 22, 23, and 24 increased considerably, and the accuracy of fold 27 showed improvement. Finally, adding the feature parameter based on predicted secondary structure information resulted in an overall accuracy of 78.38%. Above all, as relevant feature parameters were gradually added, the accuracies of a large majority of folds improved in different degrees. Thus, the feature parameters used were very effective in predicting 27-class folds. According to the previous researches [16–18, 23], we combined the training set and testing set, and the corresponding prediction results and standard deviation (in parenthesis) by 5-fold cross validation test were listed in the sixth column of Table 3. With the same dataset, the previous results of Liu et al. [27] by independent test were also listed in Table 3 for comparison. We can see that the overall accuracy by independent test in our work was 12% higher than that of Liu et al. [27] (the penultimate column in Table 3), and the overall accuracy by 5-fold cross validation test in our work was higher. The web server for protein folds prediction is accessible to the public (http://202.207.29.245:8080/Ha/HomePage/fzxHomePage.jsp).

### 3.2. The Comparison with Previous Results

To test the efficiency of our method, with the same feature parameters, classification strategy, and algorithm used above, the 27-class folds dataset built by Ding and Dubchak [11] was also tested. An overall accuracy of 70.24% was achieved by independent test (the last column in Table 3). The previous results from the same dataset are also listed in Table 4 for comparison. The accuracy was slightly lower than the results of Shen and Chou [24] and Yang et al. [25], but the overall accuracy in our work was higher than the accuracies of most previous researchers (Table 4).

### 3.3. The Identification Results of Structural Classifications

According to Shen and Chou's [21] description on structural classification in the literature, 27-class protein folds belong to four types of structural classification (Table 1). Therefore, the same feature parameters above were extracted and the combined feature parameters were inputted in Random Forest algorithm; the overall accuracy of the four types of structural classification in the testing set measured up to 92.55% by independent test. This overall accuracy was 3% higher than that of Liu et al. [26] (Table 5). Our approach was also tested on the dataset built by Ding and Dubchak which has been used by many researchers and our results were superior to most previous results derived from the same dataset (Table 5).

## 4. Conclusion

In early 2001, Ding and Dubchak built the 27-class folds dataset and started research on the identification of 27-class protein folds with multiple feature groups. Researchers have since been devoted to the improvement of feature parameters, algorithms, classification strategies, and the datasets for the identification of protein folds and have achieved good identification results. Based on previous researches, we combined sequence information, structural information, and functional information as input feature parameters of the Random Forest algorithm for protein folds identification and obtained better results. Therefore, the addition of segment interactions and motif information for recognizing 27-class protein folds is a valid and novel approach.

Given the same dataset, when different feature parameters are used, the same sequence can be correctly or falsely classified. Here, our approach achieved the improved results with the following possible reasons. First, in considering the correlation at the level of secondary structure segments, we calculate the interaction information of secondary structure segments, which reflects the segments-order and long-range correlation information of the sequence and has a major influence on protein folding. Second, in considering the local conservation of kernel structure in protein folds, motif information was extracted, including functional motifs and statistical motifs. Finally, the Random Forest algorithm is a combination classifier of convenience and high efficiency whose final classification results are decided by votes from decision trees.

In our future work, with the same parameters and classification strategy, testing methods (jackknife test, 10-fold cross validation, etc.) would be used. Different physiochemical properties also would be analyzed for the recognition of protein folds. Moreover, Precision-Recall curves or ROC curves for individual folds could be presented.

## Figures and Tables

**Table 1:** Datasets of 27-class protein folds.

Fold	Dataset built by Liu et al. [27]		Dataset built by Ding and Dubchak [11]	
	Training set	Testing set	Training set	Testing set
All *α* structural class	174	169	54	61
(1) Globin-like	14	14	13	6
(2) Cytochrome c	10	10	7	9
(3) DNA-binding 3-helical bundle	92	90	12	20
(4) 4-helical up-and-down bundle	25	24	7	8
(5) 4-helical cytokines	8	8	9	9
(6) Alpha; EF-hand	25	23	6	9
All *β* structural classes	260	254	109	117
(7) Immunoglobulin-like *β*-sandwich	86	85	30	44
(8) Cupredoxins	18	18	9	12
(9) Viral coat and capsid proteins	24	24	16	13
(10) ConA-like lectins/glucanases	18	17	7	6
(11) SH3-like barrel	41	41	8	8
(12) OB-fold	29	28	13	19
(13) Trefoil	11	10	8	4
(14) Trypsin-like serine proteases	17	16	9	4
(15) Lipocalins	16	15	9	7
*α*/*β* structural class	341	337	115	143
(16) (TIM)-barrel	93	92	29	48
(17) FAD (also NAD)-binding motif	5	5	11	12
(18) Flavodoxin-like	37	36	11	13
(19) NAD(P)-binding Rossmann-fold	17	16	13	27
(20) P-loop-containing nucleotide	74	73	10	12
(21) Thioredoxin-like	37	36	9	8
(22) Ribonuclease H-like motif	39	40	10	12
(23) Hydrolases	33	33	11	7
(24) Periplasmic binding protein-like	6	6	11	4
*α* + *β* structural class	181	179	33	62
(25) *β*-Grasp	39	39	7	8
(26) Ferredoxin-like	101	99	13	27
(27) Small inhibitors, toxins, and lectins	41	41	13	27

Overall	956	939	311	383

**Table 2:** The physicochemical property values for 20 amino acid residues.

Code	H_1_	H_2_	PL	SASA
A	0.62	−0.5	8.1	1.181
C	0.29	−1	5.5	1.461
D	−0.9	3	13	1.587
E	−0.74	3	12.3	1.862
F	1.19	−2.5	5.2	2.228
G	0.48	0	9	0.881
H	−0.4	−0.5	10.4	2.025
I	1.38	−1.8	5.2	1.81
K	−1.5	3	11.3	2.258
L	1.06	−1.8	4.9	1.931
M	0.64	−1.3	5.7	2.034
N	−0.78	2	11.6	1.655
P	0.12	0	8	1.468
Q	−0.85	0.2	10.5	1.932
R	−2.53	3	10.5	2.56
S	−0.18	0.3	9.2	1.298
T	−0.05	−0.4	8.6	1.525
V	1.08	−1.5	5.9	1.645
W	0.81	−3.4	5.4	2.663
Y	0.26	−2.3	6.2	2.368

**Table 3:** Prediction accuracies of different parameters in the testing set (%).

Fold	A	A + ACC	A + ACC + M	A + ACC + M + P	A + ACC + M + P (5-fold cross validation)	The results of Liu et al. [27]	Ding and Dubchak's dataset [11] A + ACC + M + P
1	21.43	71.43	71.43	71.43	75.00 (0.0252)	78.5	100.00
2	10.00	70.00	70.00	80.00	95.00 (0.0000)	90.0	100.00
3	60.00	90.00	91.11	91.11	92.86 (0.0026)	75.5	75.00
4	4.17	83.33	75.00	75.00	81.63 (0.0000)	54.1	87.50
5	12.50	25.00	12.50	25.00	18.75 (0.0187)	25.0	77.78
6	0.00	60.87	52.17	52.17	75.00 (0.0342)	39.1	66.67
7	87.06	91.76	89.41	90.59	89.47 (0.0114)	82.3	79.55
8	11.11	27.78	27.78	38.89	41.67 (0.0000)	55.5	75.00
9	45.83	50.00	50.00	58.33	70.83 (0.0421)	70.8	84.62
10	23.53	35.29	47.06	52.94	57.14 (0.0255)	47.0	66.67
11	24.39	56.10	48.78	58.54	70.73 (0.0185)	43.9	37.50
12	0.00	46.43	64.29	60.71	54.39 (0.0096)	60.7	89.47
13	0.00	30.00	50.00	60.00	66.67 (0.0426)	10.0	50.00
14	37.50	56.25	62.50	62.50	81.82 (0.0000)	75.0	25.00
15	53.33	40.00	40.00	46.67	67.74 (0.0136)	40.0	100.00
16	86.96	95.65	98.91	100.00	98.92 (0.0144)	89.1	66.67
17	0.00	20.00	20.00	20.00	20.00 (0.0097)	20.0	91.67
18	11.11	30.56	47.22	61.11	68.49 (0.0894)	16.6	38.46
19	37.50	81.25	100.00	100.00	100.00 (0.0300)	81.2	62.96
20	26.03	72.60	90.41	89.04	91.84 (0.0398)	87.6	41.67
21	30.56	50.00	75.00	72.22	72.60 (0.0217)	52.7	75.00
22	22.50	40.00	62.50	57.50	65.82 (0.0113)	50.0	41.67
23	27.27	45.45	90.91	90.91	95.46 (0.0107)	78.7	57.15
24	0.00	16.67	50.00	66.67	41.67 (0.0373)	50.0	25.00
25	12.82	56.41	61.54	61.54	69.23 (0.0233)	30.7	12.50
26	51.52	88.89	90.91	92.93	86.00 (0.0104)	67.6	62.96
27	100.00	75.61	87.80	92.68	92.68 (0.0122)	1.000	96.30
*Q*	43.66	68.80	76.25	**78.38**	**81.16 (0.0028)**	66.5	**70.24**

Note: A means amino acid composition (20 dimensions), A + ACC means amino acid composition and the interaction of segments (164 dimensions), A + ACC + M means amino acid composition, the interaction of segments, and motif frequency (290 dimensions), and A + ACC + M + P means amino acid composition, the interaction of segments, motif frequency, and predicted secondary structure information (296 dimensions); *Q* means the overall accuracy; the standard deviation values are in the parenthesis of the sixth column, the penultimate column is the results of Liu et al. [27] with the same dataset, and the last column is our results of the dataset built by Ding and Dubchak [11].

**Table 4:** The previous identification results by an independent test from Ding and Dubchak's dataset (%).

Author	Classifier	Accuracy
Ding and Dubchak [11]	SVM (All-Versus-All)	56.0
Chinnasamy et al. [12]	Tree-Augmented Naive Bayesian Classifier	58.2
Shen and Chou [21]	OET-KNN	62.1
Nanni [13]	Fusion of classifiers	61.1
Chen and Kurgan [22]	PFRES	68.4
Guo and Gao [14]	GAOEC	64.7
Damoulas and Girolami [15]	Multiclass multikernel	70.0
Zhang et al. [20]	Increment of diversity	61.1
Ghanty and Pal [23]	Fusion of different classifiers	68.6
Dong et al. [18]	ACCFold	70.1
Shen and Chou [24]	PFP-FunDSeqE	70.5
Yang et al. [25]	MarFold	71.7
Liu et al. [27]	SVM	69.8
**Our work**	Random Forest	**70.2**

**Table 5:** Overall accuracies of structural class using different approaches in the testing set (%).

Dataset	Author	Structural class				Accuracy
		*α*	*β*	*α*/*β*	*α* + *β*	
Liu and Hu [26]	**Our work**	**94.67**	**91.73**	**97.33**	**82.68**	**92.55**
	Liu and Hu [26]	97.04	85.43	94.07	78.21	89.24

Ding and Dubchak [11]	**Our work**	**83.60**	**88.89**	**82.52**	**66.13**	**81.98**
	Liu and Hu [26]	86.89	88.03	83.22	59.68	81.46
	Zhang et al. [20]					79.11
	Chinnasamy et al. [12]					80.52
